# Supplementary material for: Vibrational couplings and energy transfer pathways of water’s bending mode
Source: Nat Commun. 2020 Nov 25;11:5977. doi: 10.1038/s41467-020-19759-w (PMC7688972; doi:10.1038/s41467-020-19759-w)
Supplement: Supplementary file 1 — Supplementary Information [file 41467_2020_19759_MOESM1_ESM.pdf]

Supplementary Information

Vibrational Couplings and Energy Transfer Pathways of Water's Bending Mode

Yu et al.

Supplementary Figures

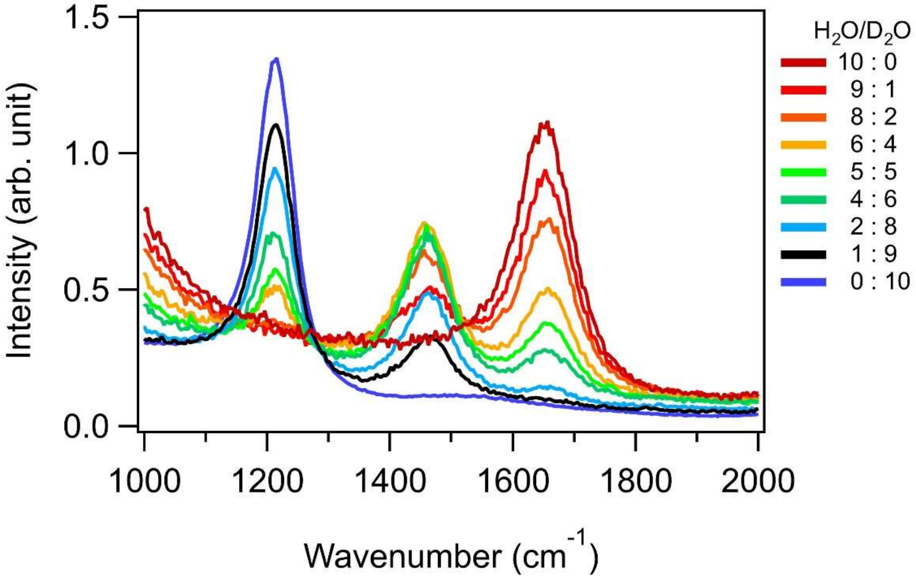

Supplementary Figure 1. FTIR spectra of various H_2O-D_2O mixtures. The legends represent the H_2O/D_2O ratios of the samples.

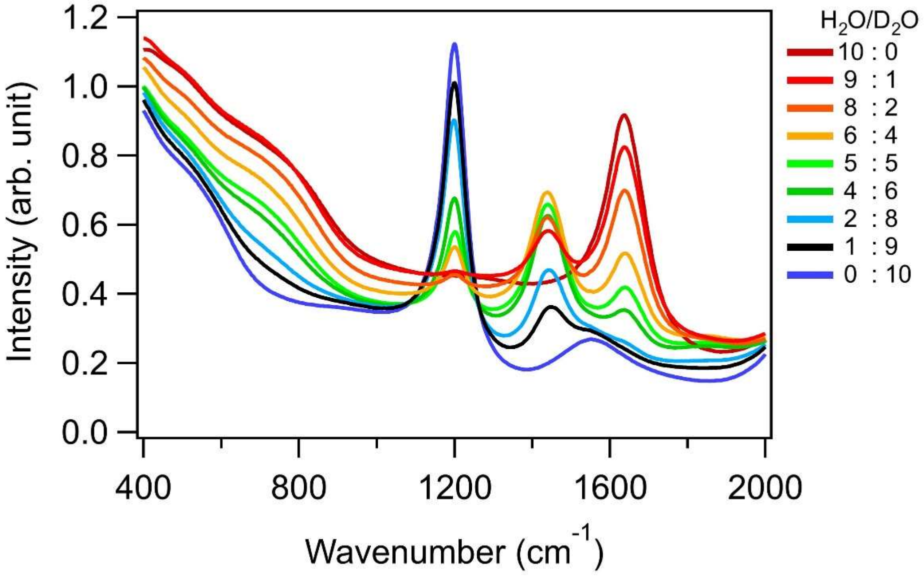

Supplementary Figure 2. Raman spectra of various H_2O-D_2O mixtures. The legends represent the H_2O/D_2O ratios of the samples.

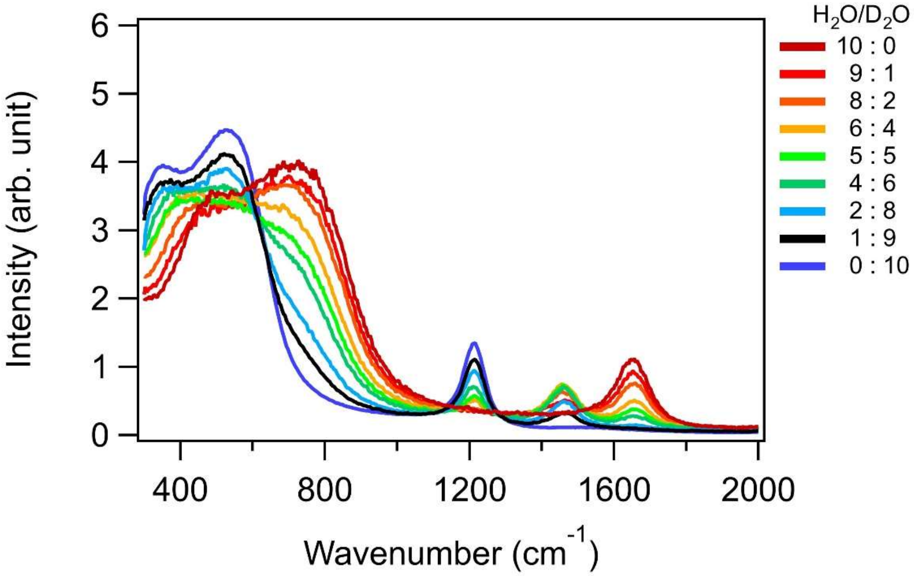

Supplementary Figure 3. Hyper-Raman spectra of various H_2O-D_2O mixtures. The legends represent the H_2O/D_2O ratios of the samples.

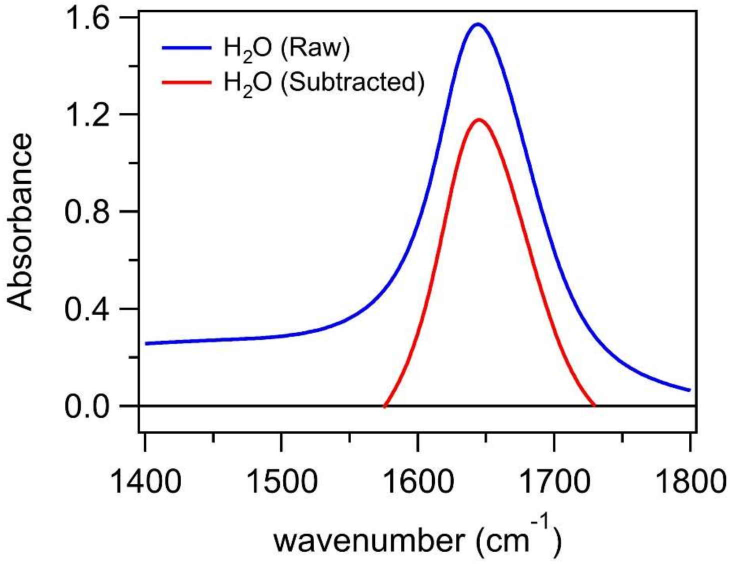

Supplementary Figure 4. The IR raw and corrected spectra of pure H_2O. The blue line represents the raw spectrum. The red line represents the corrected spectrum by subtraction for the background signal from 1575 to 1730 cm^{-1}.

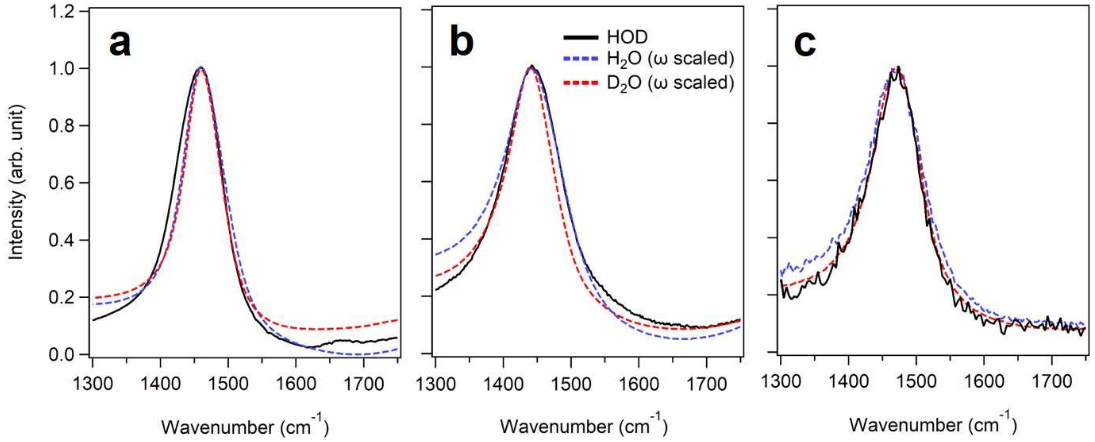

Supplementary Figure 5. The HOD spectra. **a** The FTIR, **b** Raman, and **c** hyper-Raman spectra of pure HOD deduced from Supplementary Eq. (12). We also plot the FTIR, Raman, and hyper-Raman spectra of H_2O and D_2O by scaling frequency with the factors of 0.888 and 1.207, respectively. All the spectra were normalized at the peak maximum.

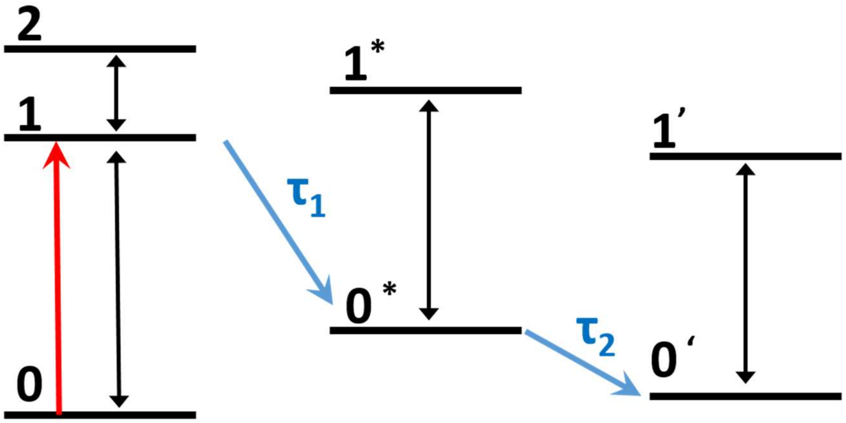

Supplementary Figure 6. Schematic representation of the kinetic model used to describe the vibrational relaxation of the H-O-H bend vibration. The excited state relaxes with a time constant τ_1 to an intermediate state, 0^*. The intermediate state subsequently relaxes to the hot ground state, 0’, with a time constant τ_2.

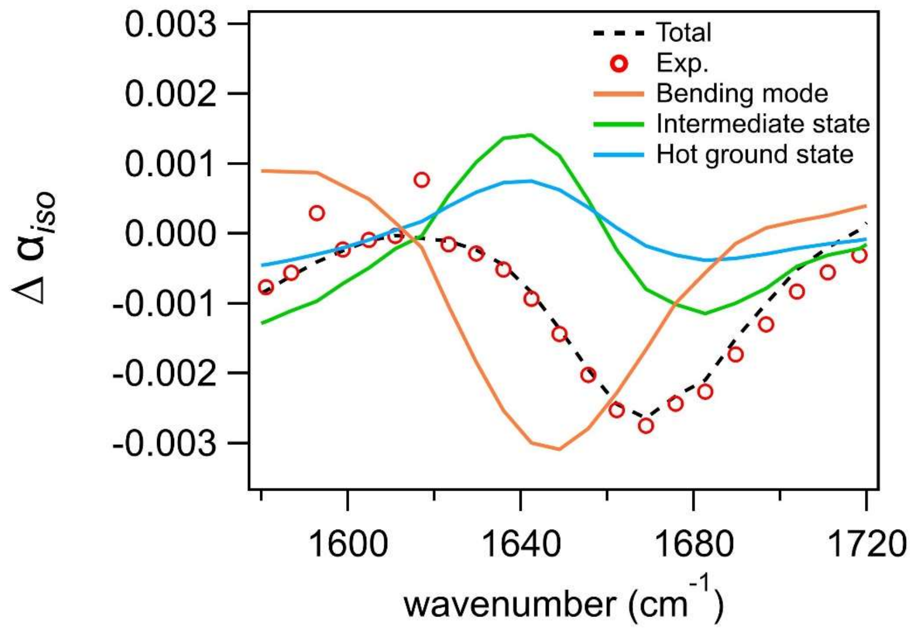

Supplementary Figure 7. The transient spectrum of the four-state model for H_2O:D_2O = 6:4 at 0.28 ps. The excited state contribution (orange line) shows typical excited state signatures: a ground state bleach at ~1640 cm^{-1} and an excited absorption at ~1580 cm^{-1}. The intermediate state (green line) and hot ground state (blue line) contributions, as obtained from the fit, exhibit similar spectra shapes, yet with different spectral amplitudes.

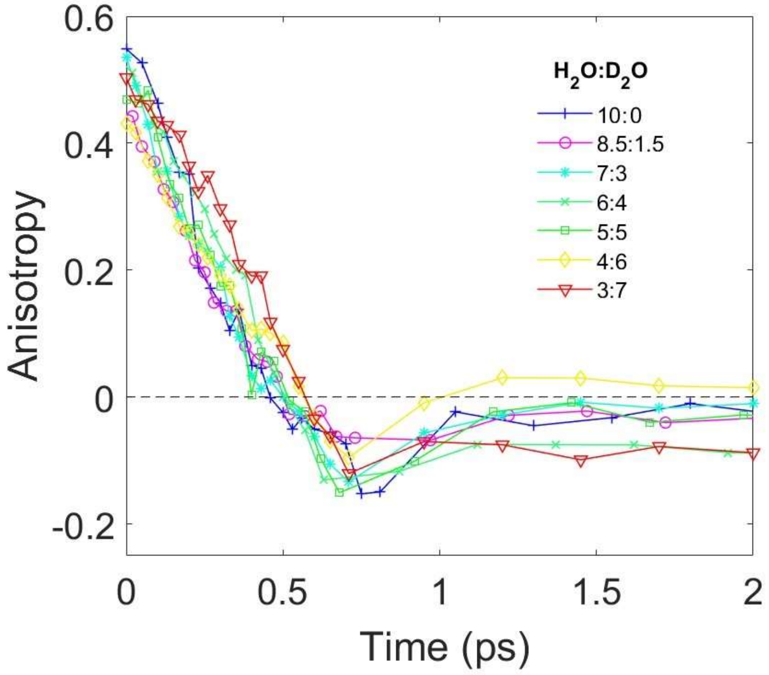

Supplementary Figure 8. Anisotropic signal integrated in the 1649 cm^{-1} < ω < 1678 cm^{-1} without heat subtraction. It shows that a negative band appears after first 0.5 ps, resulting from the thermal signals[24].

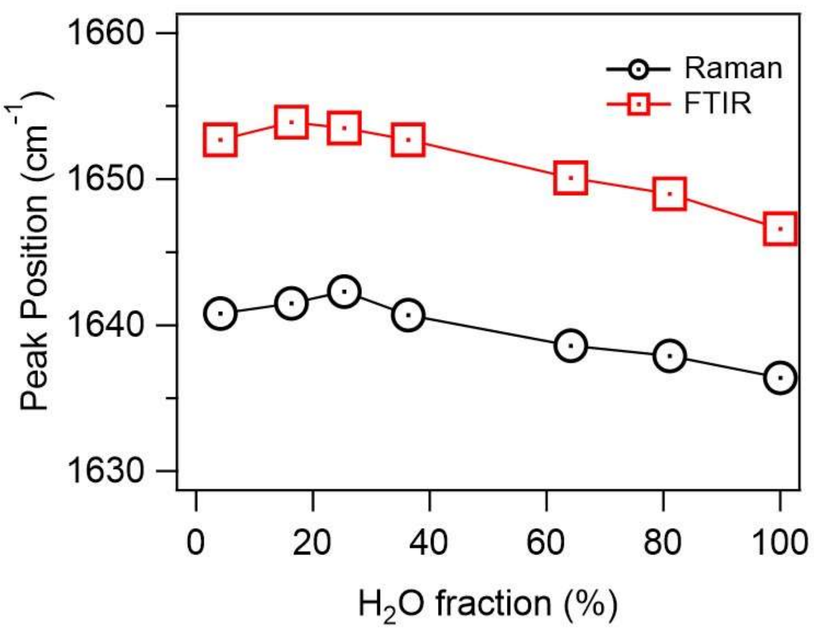

Supplementary Figure 9. The peak position for the extracted H-O-H bending mode contributions vs. H_2O concentration.

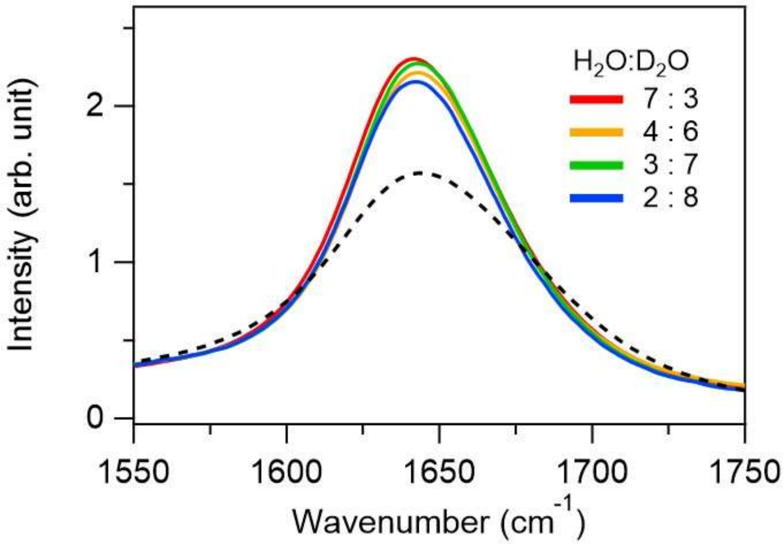

Supplementary Figure 10. Contributions of the H-O-H bending mode extracted from the measured IR spectra of 6 m $NaClO_4$ aqueous solutions with various H_2O-D_2O concentrations. The black broken lines represent the IR spectrum of pure H_2O.

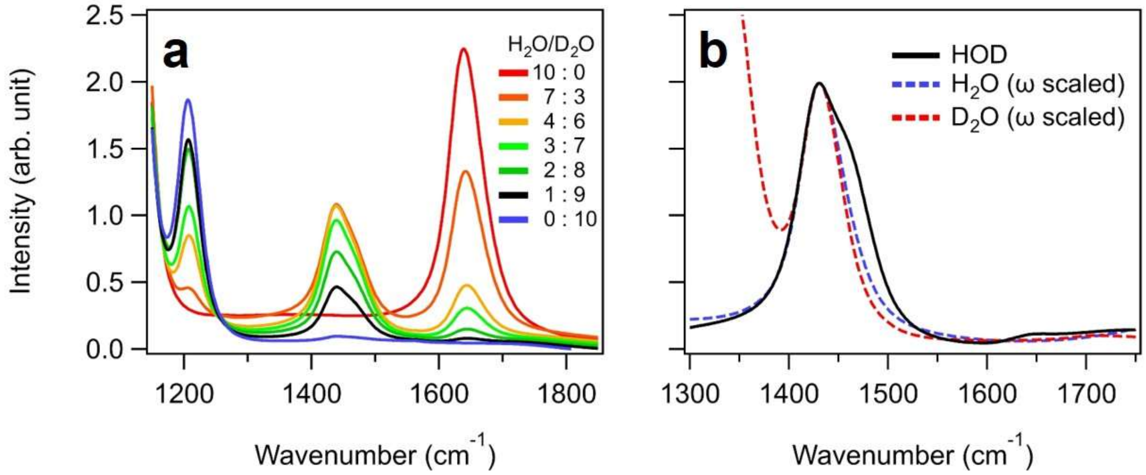

Supplementary Figure 11. The FTIR spectra of $NaClO_4$ solutions. **a** FTIR spectra of 6 m $NaClO_4$ solution with various H_2O-D_2O fractions. **b** The HOD spectrum extracted from the FTIR data of the H_2O:D_2O = 1:9 mixture. We also plot the FTIR spectra of neat H_2O and D_2O by scaling the frequency axis with the factors of 0.873 and 1.186, respectively. All spectra were normalized to the peak maximum.

Supplementary Tables

Supplementary Table 1. Coefficients for Vibrational Mode Decomposition.

Mode	c_1	c_2	c_3	c_4	c_5	c_6
bend	0.06	0.06	0.19	-0.73	-0.73	0.19
s-stretch	0.04	0.04	-0.72	0.06	0.06	-0.72
as-stretch	0.05	-0.05	-0.73	0.01	-0.01	0.73

Supplementary Notes

Supplementary Note 1: Fourier Transform Infrared (FTIR) Experiment

The isotopic mixtures were obtained by mixing the appropriate volumes of Milli-Q H_2O (18 MΩ-cm resistivity) and D_2O (99.9 atom % D, Euriso-Top). Sodium perchlorate (~3.7g, 98%, anhydrous, abcr GmbH & co.KG) were dissolved in 5 mL of H_2O or D_2O, and the salt concentration was 6 m. The isotopic mixtures were prepared by mixing these pure salt solutions in H_2O and D_2O at various volume fractions. FTIR spectra were recorded with a Bruker VERTEX 70 FTIR spectrometer in transmission. The samples were held between two CaF_2 windows separated by a 15 μm-thick Teflon spacer. The spectrometer was purged with N_2 and spectra were recorded at 298 K with a 2 cm^{-1} resolution.

Supplementary Note 2: Raman Experiment

We used ultra-pure H_2O (18 MΩ-cm resistivity, from PURELAB flex, ELGA) and D_2O (99.9 atom % D, ISOTEC). The isotopic mixtures were prepared volumetrically. Raman excitation was made with a 532 nm laser (Millennia eV, Spectra-Physics). The laser power was 100 mW. The sample liquids were contained in a quartz cuvette (1 cm × 1 cm). The Raman signal was collected in a 90-degrees scattering geometry. The Raman signal was filtered using a polarizer and a long-pass filter, before detection in a spectrograph (SR303i-B, Andor: 1200 grooves/mm) equipped with a CCD camera (DU420A-BVF, Andor). 100 spectra, each measured for 1 sec, were averaged for one sample. We measured the signal in the VV (parallel) polarization configuration.

Supplementary Note 3: Hyper-Raman Experiment

We used ultra-pure H_2O (18 MΩ-cm resistivity, from PURELAB flex, ELGA) and D_2O (99.9 atom % D, ISOTEC). The experimental setup is described in detail elsewhere.[1] Briefly, we used a picosecond laser (Cepheus 1002, Photon Energy, wavelength: 1064 nm, pulse width: ~15 ps, repetition rate: 150 kHz). The output of the laser was frequency-doubled by a BBO crystal. The 532 nm laser with ~300 mW output power was focused into the sample, which was contained in a quartz cell (10 × 10 mm^2). Hyper-Raman signals were collected in a 90-degrees scattering geometry. The signals were collimated by a lens, passed through short-pass filters, and guided into a spectrometer (iHR320, Horiba: 2400 grooves/mm). The signals were detected by a CCD camera (DU420A-OE, Andor). Multiple spectra, varying from 3 to 60, were averaged for each sample.

Supplementary Note 4: Pump-Probe IR Experiment

Pump-probe IR measurements were performed on a femtosecond Ti: Sapphire amplified laser system (Coherent Astrella, ~800 nm, ~35 fs, 1 kHz) with 6.8 W output power. 2.8 W of the output was used to pump an optical parametric amplifier (TOPAS, light conversion) with a non-collinear DFG stage to generate broadband IR pulses (centered at 1600 cm^{-1}, 100 fs duration, 17 μJ pulse energy, and 300 cm^{-1} full width at half maximum (FWHM)). The IR pulses were split into probe (~2.5%), reference (~ 2.5%), and pump pulses (~ 95%). The reference pulse was used to correct for pulse-to-pulse energy fluctuations. The time delay between the pump and probe pulses was controlled using a translational stage and the pump beam was modulated at 500 Hz for active noise suppression. The polarization of the pump-pulse was set at 45° with respect to the probe pulse polarization using a half-wave plate. The pump and probe pulses were focused and spatially overlapped in the sample using an off-axis parabolic mirror. The sample was placed between two CaF_2 windows separated by a 15 μm-thick Teflon spacer. The pump pulse was blocked after the sample and the probe pulse was re-collimated using a second parabolic mirror. A wire grid polarizer mounted in a rotating stage allowed us to select the parallel and perpendicular polarization components of the probe beam, relative to the pump polarization. Both the probe and the reference pulses were dispersed with a spectrometer onto a 2 × 32 pixel liquid-nitrogen-cooled mercury-cadmium telluride detector.

Supplementary Note 5: Simulation Protocol

We used the *ab initio* molecular dynamics (AIMD) trajectories at the revPBE0-D3(0) level of theory[2–4] as well as the classical force field molecular dynamics trajectories with the POLI2VS model[5]. The AIMD simulations employed the QUICKSTEP method[6] implemented in the CP2K program[7]. The auxiliary density matrix method (ADMM) was used[8] to reduce the computational cost for hybrid-generalized gradient approximation (GGA) calculations. We used the mixed Gaussian and plane wave approach as implemented in the CP2K code. For the Gaussian part, the TZV2P basis set was used. We set the plane wave density cutoff to 800 Ry. The norm-conserving Goedecker-Teter-Hutter pseudopotentials[9,10] were used to describe the core electrons. We used the *NPT* ensemble, where the target temperature was set to 300 K with canonical sampling through a velocity rescaling thermostat[11] and the pressure was set to 1 atm. The simulation cell contained 64 H_2O molecules. The time step was set to 0.5 fs. We prepared 8 independent samples and run the 15 ps AIMD simulation for equilibrating the systems. The equilibrated water density was 0.95 g/cm^3. Sequentially, we obtained 10 ps AIMD trajectories which were used for computing the spectra.

For the POLI2VS simulations, we carried out the *NPT* simulation at 300 K. The system temperature was controlled by the Nose-Hoover thermostat. The simulation box contained 500 H_2O molecules. The time step was set to 0.4 fs. We obtained 1 ns trajectories, from which we computed the spectra.

The VDOS spectra were calculated for the dipole moment directions via;[12,13]

$$\mathrm{VDOS}(\omega) = \int_0^T \cos(\omega t)\cos^2\left(\frac{\pi t}{2T}\right)\langle \textstyle\sum_i \mathbf{v}_i(t)\cdot\mathbf{v}_i(0)\rangle dt, \tag{1}$$

$$\mathbf{v}_i(t) = \frac{\left(v_{i,\mathrm{H1}}(t)+v_{i,\mathrm{H2}}(t)\right)}{2} - \mathbf{v}_{i,\mathrm{O}}(t), \tag{2}$$

where $\mathbf{v}_{i,x}(t)$ denotes the velocity vector of atom x=O, H_1, H_2 for water molecule i, T is the length of the time correlation function and was set to 1 ps. The VDOS spectra were decomposed based on the center of mass velocity $\mathbf{v}_{i,\mathrm{COM}}(t)$, rotational motion velocity $\mathbf{v}_{i,\mathrm{ROT}}(t)$, intramolecular bending motion velocity $\mathbf{v}_{i,\mathrm{INTRA(bend)}}(t)$, and intramolecular stretch motion velocity $\mathbf{v}_{i,\mathrm{INTRA(stretch)}}(t)$.

$$\mathbf{v}_{i,\mathrm{COM}}(t) = \left(m_\mathrm{H}\mathbf{v}_{i,\mathrm{H1}}(t) + m_\mathrm{H}\mathbf{v}_{i,\mathrm{H2}}(t) + m_\mathrm{O}\mathbf{v}_{i,\mathrm{O}}(t)\right)/(2m_\mathrm{H} + m_\mathrm{O}), \quad (3)$$

$$\mathbf{v}_{i,\mathrm{INTRA(bend)}}(t) = c_{i,\mathrm{bend}}(t)\left(-0.33\mathbf{u}_{i,\mathrm{OH1}}(t) - 0.33\mathbf{u}_{i,\mathrm{OH2}}(t)\right), \quad (4)$$

$$\mathbf{v}_{i,\mathrm{INTRA(stretch)}}(t) = \mathbf{v}_{i,\mathrm{INTRA(s-stretch)}}(t) + \mathbf{v}_{i,\mathrm{INTRA(as-stretch)}}(t), \quad (5)$$

$$\mathbf{v}_{i,\mathrm{INTRA(s-stretch)}}(t) = c_{i,\mathrm{s-stretch}}(t)\left(-0.37\mathbf{u}_{i,\mathrm{OH1}}(t) - 0.37\mathbf{u}_{i,\mathrm{OH2}}(t)\right), \quad (6)$$

$$\mathbf{v}_{i,\mathrm{INTRA(as-stretch)}}(t) = c_{i,\mathrm{as-stretch}}(t)\left(-0.42\mathbf{u}_{i,\mathrm{OH1}}(t) + 0.42\mathbf{u}_{i,\mathrm{OH2}}(t)\right), \quad (7)$$

$$\mathbf{v}_{i,\mathrm{ROT}}(t) = \mathbf{v}_i(t) - \mathbf{v}_{i,\mathrm{COM}}(t) - \mathbf{v}_{i,\mathrm{INTRA(bend)}}(t) - \mathbf{v}_{i,\mathrm{INTRA(stretch)}}(t), \quad (8)$$

where m_H and m_O denote the mass of H and O atoms, respectively. $\mathbf{u}_{i,\mathrm{OH1}}$ and $\mathbf{u}_{i,\mathrm{OH2}}$ represent the unit OH1 and OH2 vectors, respectively, of the water molecule i. $c_{i,\mathrm{mode}}(t)$, mode=bend, symmetric-stretch (s-stretch), asymmetric-stretch (as-stretch), is given as follows;

$$\begin{aligned} c_{i,\mathrm{mode}}(t) = (&c_{1,\mathrm{mode}}\mathbf{v}_{i,\mathrm{O}}(t)\cdot\mathbf{u}_{i,\mathrm{OH1}}(t) + c_{2,\mathrm{mode}}\mathbf{v}_{i,\mathrm{O}}(t)\cdot\mathbf{u}_{i,\mathrm{OH2}}(t) \\ &+ c_{3,\mathrm{mode}}\mathbf{v}_{i,\mathrm{H1}}(t)\cdot\mathbf{u}_{i,\mathrm{OH1}}(t) + c_{4,\mathrm{mode}}\mathbf{v}_{i,\mathrm{H1}}(t)\cdot\mathbf{u}_{i,\mathrm{OH2}}(t) \\ &+ c_{5,\mathrm{mode}}\mathbf{v}_{i,\mathrm{H2}}(t)\cdot\mathbf{u}_{i,\mathrm{OH1}}(t) + c_{6,\mathrm{mode}}\mathbf{v}_{i,\mathrm{H2}}(t)\cdot\mathbf{u}_{i,\mathrm{OH2}}(t))/A_\mathrm{mode} \end{aligned}$$

$$A_\mathrm{mode} = \sqrt{\left(c_{1,\mathrm{mode}}^2 + c_{2,\mathrm{mode}}^2 + c_{3,\mathrm{mode}}^2 + c_{4,\mathrm{mode}}^2 + c_{5,\mathrm{mode}}^2 + c_{6,\mathrm{mode}}^2\right)}, \quad (9)$$

where the coefficients of $c_1, c_2, \cdots, c_6$ for a specific vibrational mode were computed based on the normal mode of the water molecule in the gas phase at the revPBE0-D3(0) level of theory using the ORCA program and were summarized in Supplementary Table 1[14].

Supplementary Note 6: Extracting H-O-D Bending Mode Contribution from the Static Spectra

The spectral feature in the 1550 - 1750 cm^{-1} region arises not only from the H-O-H bending mode but also from the tail of the H-O-D bending mode centered at 1460 cm^{-1} and a combination band of D_2O centered at 1540 cm^{-1}. To discuss the H-O-H bending mode features of the isotopically diluted water, we isolated the H-O-H bending mode contribution (χ_{HOH}) via:

$$\chi_{\mathrm{HOH}}(c_{\mathrm{H2O}};\omega) = \frac{(\chi_{\mathrm{measured}}(c_{\mathrm{H2O}};\omega) - c_{\mathrm{HOD}}\chi_{100\%\mathrm{HOD}}(\omega) - c_{\mathrm{D2O}}\chi_{100\%\mathrm{DOD}}(\omega))}{c_{\mathrm{H2O}}}, \quad (10)$$

where $\chi_{100\%\mathrm{HOD}}$ and $\chi_{100\%\mathrm{DOD}}$ denotes the bending mode signature of 100% HOD and 100% D_2O, respectively, while $\chi_{\mathrm{measured}}(c_{\mathrm{HOH}};\omega)$ is the measured spectra at a given H_2O concentration, c_{H2O}. The concentrations of HOD and D_2O (denoted by c_{HOD} and c_{D2O}, respectively) were determined via:[15–17]

$$\frac{c_{\mathrm{HOD}}^2}{c_{\mathrm{H2O}}c_{\mathrm{D2O}}} = 3.86, \quad (11)$$

with $c_{\mathrm{H2O}} + c_{\mathrm{D2O}} + c_{\mathrm{HOD}} = 1$. Here, the use of $\chi_{100\%\mathrm{HOD}}$ and $\chi_{100\%\mathrm{DOD}}$ in Supplementary Eq. (10) assumes that the intermolecular couplings of the H-O-D bending modes (1460 cm^{-1}) and D-O-D bending modes (1250 cm^{-1}) do not affect the H-O-H bending mode (1650 cm^{-1}) critically. This assumption is justified by the fact that the H-O-D and D-O-D bending frequencies differ significantly from the H-O-H bending frequency.

We used 100% D_2O spectra as $\chi_{\mathrm{D2O}}(\omega)$. To obtain $\chi_{\mathrm{HOD}}(\omega)$, we used the relation as:

$$\chi_{100\%\mathrm{HOD}}(\omega) = \frac{(\chi_{\mathrm{measured}}(c_{\mathrm{HOD}};\omega) - c_{\mathrm{H2O}}\chi_{100\%\mathrm{HOH}}(\omega) - c_{\mathrm{D2O}}\chi_{100\%\mathrm{DOD}}(\omega))}{c_{\mathrm{HOD}}}, \quad (12)$$

where we used 100% H_2O and 100% D_2O spectra for χ_{HOH} and χ_{DOD}, respectively, and we used the smallest H_2O concentration data (H_2O:D_2O = 1:9 mixture), to minimize the error of Supplementary Eq. (10) in the H-O-H bending frequency region. The obtained $\chi_{100\%\mathrm{HOD}}(\omega)$ are

shown in Supplementary Fig. 5. By inserting the $\chi_{100\%\mathrm{HOD}}(\omega)$ spectra in Supplementary Eq. (10), we obtained $\chi_{\mathrm{HOH}}(c_{\mathrm{H2O}};\omega)$. The data are plotted in Fig. 2 of the main manuscript.

To check the validity of the deduced $\chi_{100\%\mathrm{HOD}}(\omega)$, we compared $\chi_{100\%\mathrm{HOD}}(\omega)$ via Supplementary Eq. (12) with $\chi_{100\%\mathrm{HOH}}(\omega)$ and $\chi_{100\%\mathrm{DOD}}(\omega)$. These are also plotted in Supplementary Fig. 5. The three spectra of the H-O-H, H-O-D, and D-O-D bending modes are in good agreement after frequency scaling, which demonstrates the robustness of the procedure to obtain the pure mode spectra, herein described.

Supplementary Note 7: Error Estimation of FWHM

In the above procedure, we deduce the pure H-O-D bending mode spectrum ($\chi_{100\%\mathrm{HOD}}(\omega)$) from the data obtained from a H_2O-D_2O mixture. Ideally, the H-O-D bending mode spectra would be deduced from the nearly 0% H_2O spectrum to minimize the effects of the H-O-H bending mode contribution in the deduced H-O-D bending mode spectra. In practice, we used the spectra of H_2O:D_2O = 1:9 for deducing the H-O-D bending mode contribution. The error bars shown in Fig. 2 d of the main manuscript were estimated by comparing the FWHM obtained from the spectra of H_2O:D_2O = 0:10 and either H_2O:D_2O = 1:9 or 4:6 mixtures to subtract the background.

To do so, we used the spectra of H_2O:D_2O = 4:6 concentration to deduce the H-O-D bending mode spectra and subsequently we obtained the FWHMs. These FWHMs are denoted as FWHM_2, while the FWHMs obtained from the deduced H-O-D bending mode spectra with H_2O:D_2O = 1:9 concentration are denoted as FWHM_1. The errors, σ, were estimated via;

$$\sigma = \frac{|\mathrm{FWHM}_1 - \mathrm{FWHM}_2|}{3}. \qquad (13)$$

The factor 3 arises from the concentration difference of H_2O between 1:9 and 4:6 (factor 3 difference) used for the error estimation. These error bars are shown in the Fig. 2d in the main text.

Supplementary Note 8: Isotropic Relaxation Data Processing

We used a four-state kinetic model (Supplementary Fig. 6) to model the isotropic transient absorption data, $\Delta\alpha_{iso}(\omega,t) = \left(\Delta\alpha_{\parallel}(\omega,t) + 2\Delta\alpha_{\perp}(\omega,t)\right)/3$. In this model, the excited state of the H-O-H bending vibration, 1, decays to an intermediate state, 0*, with a characteristic time constant τ_1. Subsequently, vibrational population of the intermediate state relaxes to the hot ground state, 0', with a time constant τ_2.

We use the intermediate state in the kinetic model to describe the delayed appearance of the spectral signatures of the hot ground state after vibrational relaxation of the bending mode excitation. This delayed appearance has also been reported in Ref. 18. The intermediate state is used as a mean to model the initial re-distribution of the bending mode energy to lower frequency modes after de-population of the bending mode excitation. In the intermediate state, the bending mode is depopulated, but the excess vibrational energy is however not yet fully equilibrated over all available modes. This equilibration is closer to the thermal equilibrium in the subsequently populated hot ground state, which – similar to the intermediate state – represents the population of a manifold of low energy states. Given that these lower frequency states are differently coupled to the experimentally interrogated bending mode, they give rise to different transient bending mode spectra.

This relaxation model is described by the following differential equations:

$$\frac{dN(t)}{dt} = \frac{-N(t)}{\tau_1}$$

$$\frac{dN^*(t)}{dt} = \frac{N(t)}{\tau_1} - \frac{N^*(t)}{\tau_2}$$

$$\frac{dN'(t)}{dt} = \frac{N^*(t)}{\tau_2}, \qquad (14)$$

where $N(t), N^*(t)$and $N'(t)$ represent the populations of the bending mode excited state (1), the intermediate state (0*), and the hot ground state (0'), respectively. Each state is assumed to

have its own characteristic transient spectrum ($\sigma(\omega)$, $\sigma^*(\omega)$, and $\sigma'(\omega)$) and the experimental spectra are modelled as the sum of these spectra multiplied by their time-dependent population:

$$\Delta\alpha_{iso}(\omega,t) = N(t)\sigma(\omega) + N^*(t)\sigma^*(\omega) + N'(t)\sigma'(\omega) \quad (15)$$

In Fig. 3 (d) of the main manuscript the integrated time traces (1649 cm^{-1} < ω < 1662 cm^{-1}) are shown.

Note that this energy relaxation model differs from the model used in Ref.18. In Ref.18, the contribution of the hot ground state to the spectra is described by a single exponential ingrowth. However, based on the time-resolved study of Ref.19, it is reasonably assumed that the bending mode vibrational energy relaxed first to the librational mode with a time constant of 170 fs, then subsequently relaxed to the hot ground state, which is accounted for in the model employed here.

Fits of Supplementary Eq. (15) to the experimental data (see Figs. 3 a and b of the main manuscript) and the temporal evolution of the contribution of the three states are exemplarily shown in Fig. 3 b of the manuscript. From these fits we obtain relaxation times τ_1~0.2 ps and τ_2~1 ps (Fig. 3 c), in good agreement with earlier studies[18,20,21]. Most importantly, these relaxation times are insensitive to the H_2O:D_2O ratio, suggesting that coupling of the bending mode to lower frequency modes is independent of isotopic composition.

Supplementary Note 9: Excitation Anisotropy

To obtain the orientational memory of the bending mode on the excitation polarization, the orientational memory of the intermediate state and the hot ground state needs to be considered. Typically, for energy relaxation on short timescales, the initially dissipated energy is distributed locally around the excited oscillator and thus the orientational memory of the excited oscillator is pertained. Only at longer timescales, when the dissipated energy also modulates more distant chromophores, which were initially not excited, the contributions of relaxed states can give rise to a decay of the anisotropy[22,23].

In our analysis, we assume that for the hot ground state the dissipated energy modulates all bending chromophores. Hence, to obtain the excitation anisotropies as shown in Fig. 4a of the main manuscript, we subtract the contribution of the hot ground state, 0', from the experimental data. This we achieve by subtracting the long time transient signal at (t = 5 ps) from both, parallel and perpendicular transient signals using the populations $N'(t)$ as obtained from the isotropic modelling.

For comparison, assuming all states (excited state, intermediate state, and hot ground state) retain the orientational excitation memory (i.e. the raw experimental anisotropies, Supplementary Fig. 8) results in faster anisotropy decays, in excellent agree with those reported for neat H_2O in an earlier study[24].

Both limiting cases show that the excitation anisotropy decays faster than what would be expected based on the rotational motion of water. Due to the fast vibrational relaxation (short τ_1), the obtained anisotropies $R(t)$ somewhat depend on the exact procedure how the contribution of 0' is subtracted (e.g. exact location of $t = 0$, functional form of $N'(t)$).

The anisotropy decay can be perturbed by the frequency-dependence of the transition dipole moment[25]. This non-Condon effect is pronounced, for example, for the anisotropy decay of

the O-H stretch mode. However, the non-Condon effects are negligible in the H-O-H bending mode[26]. Thus, we analyzed the anisotropy decay of the H-O-H bending mode within the Condon approximation.

Supplementary Note 10: Variation of Peak Positions with H_2O Concentration

The peak positions for the extracted H-O-H bending mode contributions with various $H_2O:D_2O$ concentration were extracted from the FTIR and Raman spectra. The data show that the peak frequency is slightly redshifted ($\Delta\nu < 8\ cm^{-1}$) with increasing H_2O concentration (Supplementary Fig. 9), in both the FTIR and Raman spectra.

Supplementary Note 11: FTIR Spectra of Salt Solutions

In the FTIR spectra of aqueous $NaClO_4$ solutions, the vibrational frequency of the H-O-H bending is lower than for pure water (Supplementary Fig. 10). The red-shift of the bending mode peak frequency upon addition of the salts to water demonstrates weakening of the hydrogen bonds in water. In the FTIR spectra of $NaClO_4$ solutions with various H_2O-D_2O compositions (Supplementary Fig. 11), the peak position and FWHM are insensitive to the H_2O concentration ($\Delta\nu < 5$ cm^{-1}; ΔFWHM < 10 cm^{-1}). Thus, the insensitivity of the FWHM to isotopic dilution, as reported for neat water in the main manuscript, also holds for aqueous $NaClO_4$ solutions, for which water's hydrogen-bond network markedly differs from neat water.

Supplementary References

1. Okuno, M. Hyper-Raman spectroscopy of polar liquids excited at 1064 nm: Acetone, acetonitrile, chloroform, and dimethyl sulfoxide. *J. Chem. Phys.* **152**, 174202 (2020).
2. Adamo, C. &Barone, V. Toward reliable density functional methods without adjustable parameters: The PBE0 model. *J. Chem. Phys.* **110**, 6158–6170 (1999).
3. Zhang, Y. &Yang, W. Comment on "generalized gradient approximation made simple". *Phys. Rev. Lett.* **80**, 890 (1998).
4. Grimme, S., Antony, J., Ehrlich, S. &Krieg, H. A consistent and accurate ab initio parametrization of density functional dispersion correction (DFT-D) for the 94 elements H-Pu. *J. Chem. Phys.* **132**, 154104 (2010).
5. Hasegawa, T. &Tanimura, Y. A polarizable water model for intramolecular and intermolecular vibrational spectroscopies. *J. Phys. Chem. B* **115**, 5545–5553 (2011).
6. Vandevondele, J. *et al.* Quickstep: Fast and accurate density functional calculations using a mixed Gaussian and plane waves approach. *Comput. Phys. Commun.* **167**, 103–128 (2005).
7. Hutter, J., Iannuzzi, M., Schiffmann, F. &VandeVondele, J. cp2k: atomistic simulations of condensed matter systems. *Wiley Interdiscip. Rev. Comput. Mol. Sci.* **4**, 15–25 (2014).
8. Guidon, M., Hutter, J. &VandeVondele, J. Auxiliary Density Matrix Methods for Hartree−Fock Exchange Calculations. *J. Chem. Theory Comput.* **6**, 2348–2364 (2010).
9. Kropman, M. F., Nienhuys, H.-K., Woutersen, S. &Bakker, H. J. Vibrational Relaxation and Hydrogen-Bond Dynamics of HDO:H_2O. *J. Phys. Chem. A* **105**, 4622–4626 (2001).
10. Goedecker, S., Teter, M. &Hutter, J. Separable dual-space Gaussian pseudopotentials. *Phys. Rev. B* **54**, 1703–1710 (1996).
11. Bussi, G., Donadio, D. &Parrinello, M. Canonical sampling through velocity rescaling. *J. Chem. Phys.* **126**, 014101 (2007).

12. Usui, K. *et al.* Ab Initio Liquid Water Dynamics in Aqueous TMAO Solution. *J. Phys. Chem. B* **119**, 10597–10606 (2015).

13. Nagata, Y., Yoshimune, S., Hsieh, C., Hunger, J. &Bonn, M. Ultrafast Vibrational Dynamics of Water Disentangled by Reverse Nonequilibrium Ab Initio Molecular Dynamics Simulations. *Phys. Rev. X* **5**, 021002 (2015).

14. Neese, F. The ORCA program system. *Wiley Interdiscip. Rev. Comput. Mol. Sci.* **2**, 73–78 (2012).

15. Meija, J., Mester, Z. &D'Ulivo, A. Mass Spectrometric Separation and Quantitation of Overlapping Isotopologues. H_2O/HOD/D_2O and H_2Se/HDSe/D_2Se Mixtures. *J. Am. Soc. Mass Spectrom.* **17**, 1028–1036 (2006).

16. Wolfsberg, M., Massa, A. A. &Pyper, J. W. Effect of vibrational anharmonicity on the isotopic self-exchange equilibria $H_2X+D_2X=2HDX$. *J. Chem. Phys.* **53**, 3138–3146 (1970).

17. Duplan, J. C., Mahi, L. &Brunet, J. L. NMR determination of the equilibrium constant for the liquid H_2O-D_2O mixture. *Chem. Phys. Lett.* **413**, 400–403 (2005).

18. Carpenter, W. B., Fournier, J. A., Biswas, R., Voth, G. A. &Tokmakoff, A. Delocalization and stretch-bend mixing of the HOH bend in liquid water. *J. Chem. Phys.* **147**, 084503 (2017).

19. Huse, N., Ashihara, S., Nibbering, E. T. J. &Elsaesser, T. Ultrafast vibrational relaxation of O-H bending and librational excitations in liquid H_2O. *Chem. Phys. Lett.* **404**, 389–393 (2005).

20. Larsen, O. F. A. &Woutersen, S. Vibrational relaxation of the H_2O bending mode in liquid water. *J. Chem. Phys.* **121**, 12143–12145 (2004).

21. Piatkowski, L. &Bakker, H. J. Vibrational dynamics of the bending mode of water interacting with ions. *J. Chem. Phys.* **135**, 214509 (2011).

22. Liu, L., Hunger, J. &Bakker, H. J. Energy relaxation dynamics of the hydration complex of hydroxide. *J. Phys. Chem. A* **115**, 14593–14598 (2011).

23. Mazur, K., Bonn, M. &Hunger, J. Hydrogen bond dynamics in primary alcohols: A femtosecond infrared study. *J. Phys. Chem. B* **119**, 1558–1566 (2015).

24. Chuntonov, L., Kumar, R. &Kuroda, D. G. Non-linear infrared spectroscopy of the water bending mode: Direct experimental evidence of hydration shell reorganization? *Phys. Chem. Chem. Phys.* **16**, 13172–13181 (2014).

25. Lin, Y. S., Pieniazek, P. A., Yang, M. &Skinner, J. L. On the calculation of rotational anisotropy decay, as measured by ultrafast polarization-resolved vibrational pump-probe experiments. *J. Chem. Phys.* **132**, 174505 (2010).

26. Ni, Y. &Skinner, J. L. IR and SFG vibrational spectroscopy of the water bend in the bulk liquid and at the liquid-vapor interface, respectively. *J. Chem. Phys.* **143**, 014502 (2015).
